# Stress-Based High-Throughput Screening Assays to Identify Inhibitors of Cell Envelope Biogenesis

**DOI:** 10.3390/antibiotics9110808

**Published:** 2020-11-13

**Authors:** Maurice Steenhuis, Corinne M. ten Hagen-Jongman, Peter van Ulsen, Joen Luirink

**Affiliations:** Department of Molecular Microbiology, Amsterdam Institute of Molecular and Life Sciences (AIMMS), Vrije Universiteit, De Boelelaan 1085, 1081 HV Amsterdam, The Netherlands; m.steenhuis@vu.nl (M.S.); c.jongman@vu.nl (C.M.t.H.-J.); j.p.van.ulsen@vu.nl (P.v.U.)

**Keywords:** *Escherichia coli*, high-throughput screening, antibiotics, potentiators, SigmaE, heat-shock, Cpx, Rcs

## Abstract

The structural integrity of the Gram-negative cell envelope is guarded by several stress responses, such as the σ^E^, Cpx and Rcs systems. Here, we report on assays that monitor these responses in *E. coli* upon addition of antibacterial compounds. Interestingly, compromised peptidoglycan synthesis, outer membrane biogenesis and LPS integrity predominantly activated the Rcs response, which we developed into a robust HTS (high-throughput screening) assay that is suited for phenotypic compound screening. Furthermore, by interrogating all three cell envelope stress reporters, and a reporter for the cytosolic heat-shock response as control, we found that inhibitors of specific envelope targets induce stress reporter profiles that are distinct in quality, amplitude and kinetics. Finally, we show that by using a host strain with a more permeable outer membrane, large-scaffold antibiotics can also be identified by the reporter assays. Together, the data suggest that stress profiling is a useful first filter for HTS aimed at inhibitors of cell envelope processes.

## 1. Introduction

Gram-negative bacteria are particularly refractory to the development of antibiotics as they have a complex, rather impermeable cell envelope that consists of two membranes: the cytosolic or inner membrane (IM) and the outer membrane (OM) separated by the aqueous periplasm. The periplasm harbors a thin mesh-like peptidoglycan (PG) layer that is connected to the OM to provide shape and protection against osmotic stress. The OM is an asymmetric bilayer composed of phospholipids in the inner leaflet and lipopolysaccharides (LPS) in the outer leaflet. The OM also contains outer membrane proteins (OMPs) that mostly comprise a β-barrel structure. The channels within the porins, which are an abundant class of OMPs, allow for the passage of smaller nutrients and waste products (<600 Da). However, the OM serves as a major barrier for larger molecules, including large-scaffold antibiotics that are effective against Gram-positive bacteria, such as vancomycin [1]. In addition, the dense and charged polysaccharide layer formed by the surface exposed LPS molecules provides a physical barrier to smaller hydrophobic antibacterial compounds. Even when antibiotics do pass the cell envelope they may be subject to drug efflux pumps that transport antibiotics out of the cell [2]. Combined, these characteristics contribute to the challenge of finding and developing novel antibiotics against Gram-negative bacteria. Specifically, target-based screening approaches have been met with limited success as targets are often intracellular and many hits were shown to be unable to reach their target in vivo [3].

On the other hand, the intricate and unique architecture of the cell envelope makes it an attractive target for antibiotics. In addition to assembly of the PG layer, the target of many established antibiotics, other underexplored processes in the cell envelope are essential for survival including the biogenesis of lipoproteins, OMPs and the LPS layer. Inhibitors of these processes may be useful as standalone antibacterials, but even if they have limited activity as such, they may disrupt the integrity of the OM sufficiently to allow the passage of larger and relatively hydrophobic antibiotics.

Bacteria have evolved elaborate extracellular stress response systems to detect and respond to changing environmental conditions that compromise the cell envelope [4]. The best characterized stress systems are the σ^E^ and Cpx response [5], which have different activating cues, but partially overlapping regulons [4]. Stress factors that compromise the cell envelope and interfere with OMP folding activate the σ^E^ stress system. Cpx responds to a variety of other signals, including misfolded periplasmic proteins, changes in lipid composition of the IM and OM and metabolic changes [5]. The σ^E^ response is mediated by transcriptional regulation of target genes through the sigma factor σ^E^. Cpx is composed of a two-component system CpxAR in which CpxA functions as histidine kinase sensor in the IM and CpxR as response regulator of the transcription of target genes.

**Figure 1:**
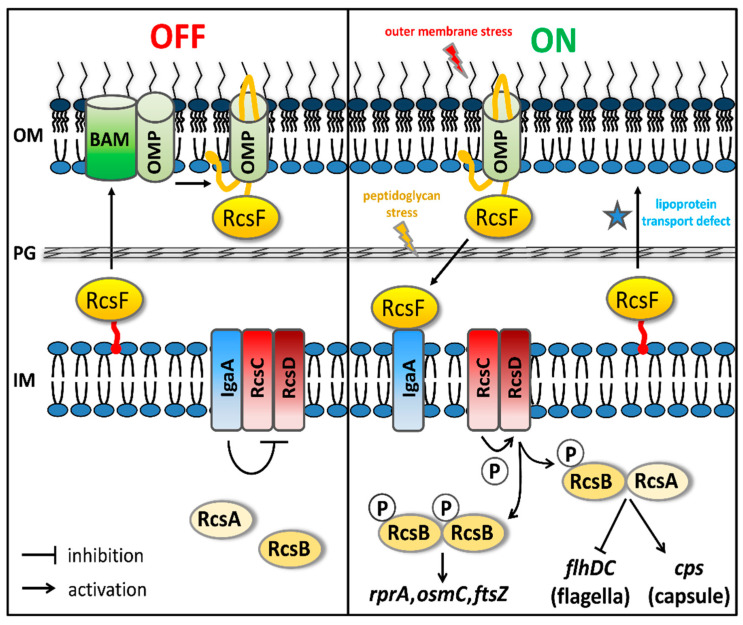
Overview of the Rcs cell envelope stress response. Please see the text for detailed information on the Rcs signal transduction cascade.

Similar to Cpx, the recently identified Rcs (regulation of capsular polysaccharide synthesis) response is built around a two-component system but with a more intricate regulatory mechanism. It is activated by perturbation of PG assembly, defective lipoprotein biogenesis and trafficking, impaired functioning of the β-barrel assembly (BAM) complex and changes in LPS charge distribution [6]. Although the underlying molecular mechanism is not yet fully elucidated, many inducing cues are signaled through the sensor protein RcsF, which is a surface-exposed OM lipoprotein (Figure 1). Under normal conditions RcsF is transported to the OM where it is threaded through the β-barrel of the abundant OMPs OmpF and OmpC by the BAM-complex during insertion into the OM [7,8]. The RcsF lipid moiety remains anchored to the inner leaflet of the OM but its main protein domain is cell-surface exposed and can directly sense changes in the LPS structure. Under stress conditions that affect its trafficking to the OM, RcsF engages the IM protein IgaA. This interaction relieves the IgaA-mediated inhibition of the RcsC sensor kinase, resulting in the sequential phosphorylation RcsC and RcsD in the IM. Subsequently, the response regulator RcsB in the cytosol is phosphorylated to act as a transcription regulator of the Rcs regulon, as a RcsB homodimer or as a heterodimer with RcsA. Effects of the Rcs stress response include increased capsule production, biofilm formation and decreased motility.

Considering the stressors of the σ^E^, Rcs and Cpx systems we reasoned that monitoring these responses in *E. coli* cells may help to identify novel agents that affect diverse aspects of cell envelope biogenesis and integrity. Recently, we described fluorescence-based high-throughput screening (HTS) assays that report on σ^E^ cell envelope stress and cytosolic heat-shock stress as a control [9]. In the current study, we developed similar assays that signal activation of the Rcs and Cpx responses. We demonstrate that perturbations of specific cell envelope processes produce unique σ^E^, Rcs, Cpx and heat-shock reporter profiles that can be exploited for drug screening purposes. In particular, we show induction of the Rcs reporter by compounds that target PG biosynthesis, lipoprotein maturation, BAM complex activity or LPS integrity. Importantly, by comparing the amplitude and kinetics of distinct reporter outputs in response to the stressors tested, we demonstrate that individual antibacterial compounds produce unique reporter profiles that could be relevant for target validation. Finally, we show that the use of the reporter assays in strains with a more permeable OM may allow the identification of large-scaffold antibiotics that normally do not cross the OM.

## 2. Materials and Methods

### 2.1. Strains and Media

The bacterial strains and plasmids that were used in this study are listed in Tables S1 and S2, respectively. *E. coli* bacteria were grown in M9 minimal medium supplemented with 0.2% glucose and 0.2% casamino acids (Difco). For selective growth chloramphenicol (30 μg/mL), kanamycin (50 μg/mL) and ampicillin (100 μg/mL) were added to the medium, where appropriate.

### 2.2. Materials, Reagents and Enzymes

A Rapid DNA Dephosphorylation & Ligation Kit was purchased from Roche Applied Science (Penzberg, Germany). Restriction enzymes were from New England Biolabs (Ipswich, MA, USA). Phusion High Fidelity DNA polymerase was from NEB and GeneJET Plasmid Miniprep Kit was from Thermo Fisher Scientific (Waltham, MA, USA). QIAquick Gel Extraction Kit and QIAquick PCR Purification Kit were from Qiagen (Hilden, Germany). The 96-well µClear Chimney black clear-bottom TC sterile plates were from Greiner Bio-One (Alphen aan den Rijn, The Netherlands). All other reagents, primers and chemicals were supplied by Sigma-Aldrich (Saint Louis, MO, USA).

### 2.3. Plasmid Construction

To construct pUA66 with the stress promoters P*rprA* and P*cpxP* fused to the gene encoding *mneongreen* (mNG) [10], the promoter region of *rprA* and *cpxP* were amplified by PCR using pUC66-RprA-GFPmut (kindly provided by Tanneke den Blaauwen) and genomic DNA of the *E. coli* strain MG1655 as template, respectively (*rprA*; FW: TCGACTCGAGAATTGATATTTGCTTGCTCTTCC, RV: GCAGGATCCGAGCTAATAGTAGGCATACGGAC, *cpxP*; FW: CTCGAGAGACGTCGCTAATCCATGAC, RV: CGTTGAATCGCGACAGAAAGAGGATCCT). The primers were flanked by *Xho*I and *Bam*HI restriction sites and the resulting PCR fragments were cloned into the *Xho*I/*Bam*HI cut pUA66 already containing *mNG*, creating pUA66-PrprA-mNG and pUA66-PcpxP-mNG. The sequences of the plasmids were confirmed by automated DNA sequencing (Macrogen Europe).

### 2.4. DjlA and Hbp Expression

For expression of DjlA, *E. coli* TOP10F’ cells, harboring pBAD22-DjlA and the compatible pUA66-PrprA-mNG, were grown in M9 at 37 °C in regular culture flasks to an OD_600_ of 0.5. The culture was then diluted to an OD_600_ of 0.1 and 50 µL aliquots were transferred to a 96-well plate that already contained 50 µL M9 and protein expression was induced with 1, 4 and 16 µM l-rhamnose (final concentration). Growth was continued at 37 °C in the Synergy H1 plate reader with 3 mm continuous linear shaking. The OD_600_ and fluorescence (485/535 nm) was measured every 15 min for 2 h.

Wild-type Hbp and Hbp110C/348C were expressed from pEH3 in *E. coli* TOP10F’ and stress was measured in the same cells with the compatible pUA66-PcpxP-mNG. Cells were grown and analyzed using the same growth regime as described previously for DjlA, except that 40 µM IPTG (final concentration) was used to induce protein expression instead of l-rhamnose.

### 2.5. Susceptibility to Antibiotics and Stress Activation

After overnight growth the bacterial cells were diluted in M9 in regular culture flasks and grown at 37 °C to mid-log phase. Then, the culture was diluted to an optical density (OD) at 600 nm of 0.1 and 50 µL culture aliquots were transferred to the wells of a black clear-bottom 96-well plate already containing 50 µL M9 with a two-fold increasing concentration of an antibacterial agent. As a control, 50 µL sterile M9 or 50 µL M9 with DMSO (0.5% as final concentration) was used (depending on the antibacterial agent, see Table 1). After sealing, the plate growth was continued in the Synergy H1 or Synergy HTX plate reader (Biotek) at 37 °C with 3 mm continuous linear shaking. Growth was determined by measuring the OD_600_ and expression of mNG by measuring the corresponding fluorescence (excitation 485 nm and emission 535 nm) every 15 min. The Z’ factor of the Rcs reporter assay was determined using an interleaved-signal model [11], using the following formula:

(1) $$Z^{\prime }=1-\left(\frac{3\sigma_{neg}+3\sigma_{pos}}{\mu_{pos}-\mu_{neg}}\right)$$

where $\sigma_{neg}$ and $\sigma_{pos}$ are defined as the calculated standard deviations of the OD_600_ corrected fluorescence values of the negative and positive controls within a plate, and $\mu_{neg}$ and $\mu_{pos}$ are the plate-averaged OD_600_ corrected fluorescence values of the negative and positive control, respectively.

## 3. Results

### 3.1. Development of Rcs and Cpx Stress Reporter Assays

We have shown previously that the σ^E^ and heat-shock stress responses in *E. coli* cells can be monitored by placing the gene encoding the green-fluorescent protein mNeonGreen (mNG) in pUA66 under control of the stress-regulated *rpoE* or *groES* promoter, respectively [9]. To monitor Rcs and Cpx stress in *E. coli* we used the same strategy and placed the mNG-encoding gene under control of the *rprA* or *cpxP* promoter, respectively.

**Figure 2:**
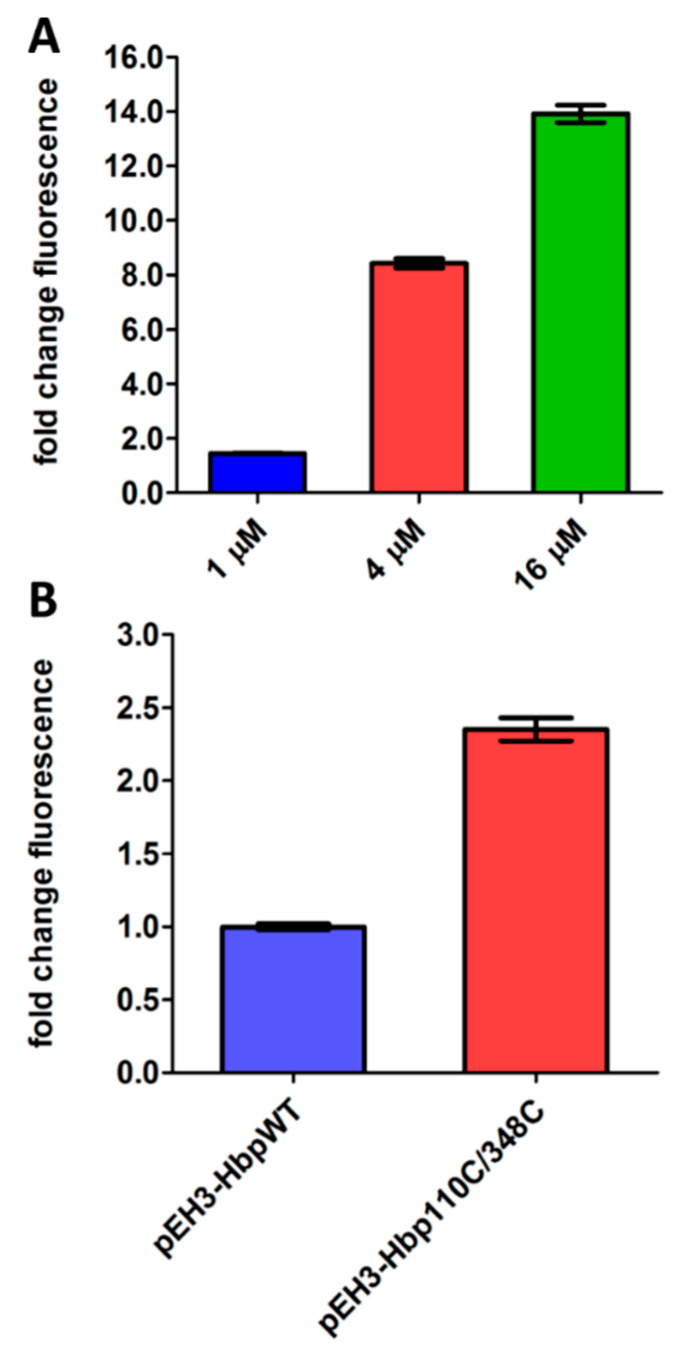
Activation of the Rcs and Cpx reporter assay using known stressors. (**A**) *E. coli* TOP10F’ cells were grown in a 96-well plate and DjlA was expressed from pBAD22 using different concentrations of L-arabinose for induction, as indicated. The Rcs response was determined using the compatible P*rprA*-mNG reporter construct in the same cells. mNG fluorescence was measured after 2 h, corrected for growth (OD_600_) and plotted as fold-change of signal compared to untreated cells (set to 1). (**B**) *E. coli* TOP10F’ cells, either containing pEH3-Hbp or pEH3-Hbp110C/348C, were grown in a 96-well plate and protein expression was induced with 40 µM IPTG. The Cpx response was determined using the compatible P*cpxP*-mNG reporter construct in the same cells. After 2 h, mNG fluorescence was measured, corrected for growth (OD_600_) and plotted as fold-change of signal compared to cells expressing Hbp-WT (set to 1). Error bars represent the standard deviation of duplicate samples. The figure shows a representative example of three independent experiments.

RprA is a small regulatory RNA that controls expression of the stationary phase σ factor *rpoS* and is regulated by the homodimeric form of phosphorylated RcsB (Figure 1) [6]. To determine whether induction of the Rcs stress response can be reliably monitored using the P*rprA*-mNG reporter construct, we examined its response to overexpression of DjlA, an IM protein with a C-terminal DnaJ-like domain [24]. Overexpression of DjlA induces Rcs stress in an RcsF-independent manner, possibly through a direct effect on IgaA or the phosphorelay system in the OM [25]. To test this, pBAD22-DjlA was introduced in *E. coli* cells together with the P*rprA*-mNG reporter construct and expression of DjlA was induced with L-arabinose. As shown in Figure 2A, the mNG fluorescence signal increased upon induction of DjlA expression in a dose-dependent manner, suggesting that the P*rprA*-mNG construct can be used to monitor Rcs stress activation.

To monitor Cpx stress, the promotor of *cpxP* that encodes a repressor of the Cpx regulon was coupled to the mNG-encoding gene. To test the resulting P*cpxP*-mNG reporter construct it was transformed into *E. coli* cells together with pEH3-Hbp110C/348C that encodes a translocation incompetent derivative of the autotransporter hemoglobin protease (Hbp) [26]. We have shown previously that Hbp110C/348C accumulates in the periplasm inducing Cpx stress response [9,26]. Indeed, as shown in Figure 2B, expression of Hbp110C/348C increased fluorescence by ~2.5 fold compared to cells expressing wild-type Hbp, indicating that this reporter construct can be used to detect Cpx activation.

### 3.2. Compromised LPS Integrity Induces Rcs and Cpx Stress Reporters

**Figure 3:**
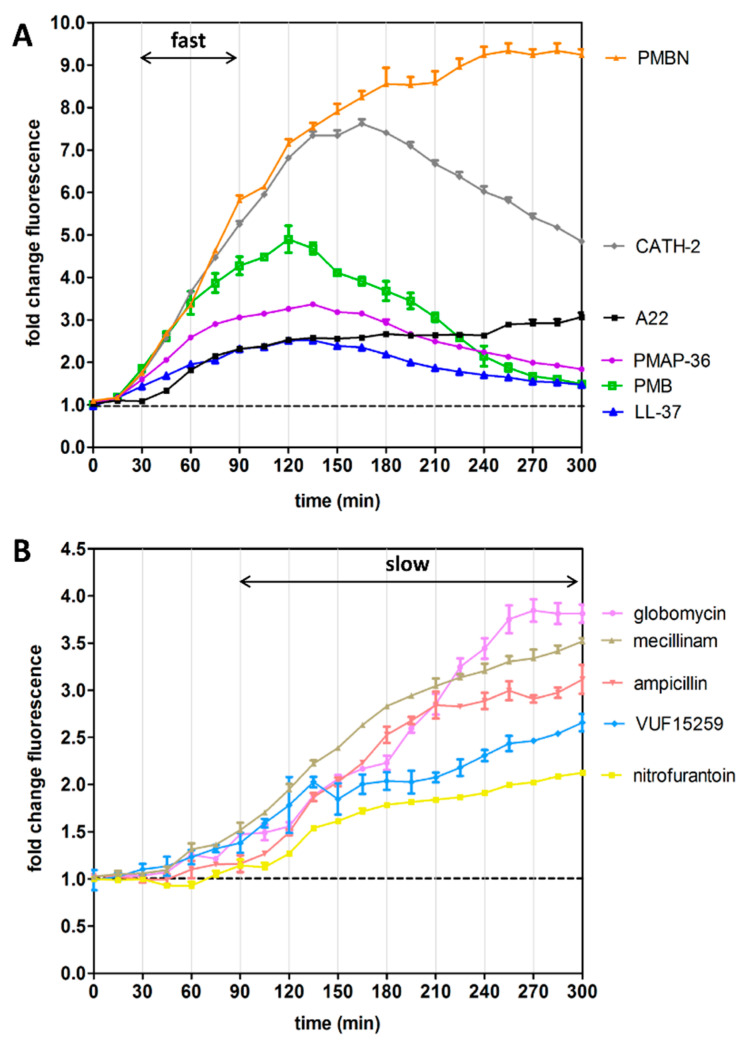
Real-time monitoring of Rcs stress activation in response to selected agents. The graph displays the kinetics of the Rcs response upon addition of the antibacterial agents that are listed in Table 1. *E. coli* TOP10F’ cells, harboring the P*rprA*-mNG reporter construct, were grown in a 96-well plate and exposed to 0.5× MIC of the indicated agents. mNG fluorescence was measured in time, corrected for growth (OD_600_) and plotted as fold-change of signal compared to untreated cells (set to 1, dashed line), with agents that show (**A**) a fast response and (**B**) a slow response, as indicated. Error bars represent the standard deviation of duplicate samples. The figure shows a representative example of three independent experiments.

To examine how *E. coli* cells respond to antibacterial agents with known targets in the cell envelope and cytosol we monitored activation of the Rcs, σ^E^ and Cpx stress responses using the P*rprA*-mNG, P*rpoE*-mNG and P*cpxP*-mNG reporters, respectively. To obtain insight in the specificity of the reactions to different cues we also monitored induction of the cytosolic heat-shock stress response using a P*groES*-mNG reporter construct [9]. *E. coli* cells harboring the reporter constructs were grown in 96-well plates containing a two-fold increasing concentration of the antibacterial agents that are listed in Table 1. After addition of agents, fluorescence and optical density at 600 nm (OD_600_) were determined to monitor stress and growth in real time. The induction of stress at 0.5× MIC (half of the minimal inhibitory concentration, see Table 1), a concentration that causes slight growth defects and stress without killing the cells, is summarized in Table 1 and is displayed in OD-corrected fluorescence units in Figure 3 and Figure S1.

To investigate induction of stress reporters upon disruption of LPS, we determined the effect of the cationic antimicrobial peptides (AMPs) polymyxin B (PMB), LL-37, PMAP-36 and cathelicidin-2 (CATH-2) that are known to interact with the negatively charged LPS and to increase OM permeability [27]. As expected, the Rcs stress response was induced by these peptides. LL-37 and PMAP-36 also induced Cpx stress but activated neither σ^E^ nor heat-shock responses. In contrast, the PMB derivative polymyxin B nonapeptide (PMBN) elicited a more generic cell envelope stress response (i.e., Rcs, σ^E^ and Cpx) but not a heat-shock response. Interestingly, PMBN lacks the fatty acid tail of PMB and has no bactericidal activity but it still binds to LPS and retains OM-permeabilizing activity [28].

Since the AMPs tested all affected cell envelope integrity and activated Rcs, and in some cases Cpx and σ^E^ stress responses, we wondered whether OM permeabilization is sufficient to activate our stress reporters. To test this, we examined the effect of ethylenediaminetetraacetic acid (EDTA) and sodium dodecyl sulfate (SDS), which both increase OM permability [1]. Additionally, we tested the effect of triclosan, which impairs OM integrity by inhibiting the activity of the enoyl-acyl carrier protein reductase (FabI), a critical enzyme in bacterial fatty acid biosynthesis [29]. After exposing cells to a sub-lethal concentration of the indicated agents, no induction of stress responses was evident, except for EDTA, which induced a Cpx stress response.

Taken together, the data suggest that it is the interaction of the AMPs with LPS rather than the permeabilization of the OM per se that is sensed by the stress systems. For the Rcs system this fits with the notion that the surface-exposed RcsF sensor can directly detect changes in the LPS layer by OM-targeting agents [15].

### 3.3. Inhibition of Biogenesis of PG, Lipoprotein and OMP Predominantly Activates the Rcs Stress Reporter

To examine PG-related stress responses, we analyzed the effect of two β-lactam antibiotics, ampicillin and mecillinam. Consistent with other studies [12], ampicillin and mecillinam induced the Rcs stress reporter, while heat-shock, Cpx and σ^E^ stress were not induced (Table 1, Figure 3 and Figure S1).

To test the effects of impaired lipoprotein biogenesis, cells were incubated with globomycin, which inhibits the lipoprotein specific signal peptidase LspA [30]. A strong induction of the Rcs and Cpx reporters was observed, again consistent with previous studies [15,16,17], while the σ^E^ and heat-shock reporters remained unaffected. Most likely, globomycin prevents maturation of RcsF and its stalling in the IM may directly relieve the IgaA-imposed negative regulation of the Rcs phosphorelay system [15,25]. How the Cpx response is induced remains to be investigated.

Finally, we investigated the consequences of perturbation of the BAM complex, which plays an essential role in folding and membrane insertion of β-barrel OMPs. In *E. coli*, the BAM complex consists of an essential β-barrel insertase (BamA) and five accessory lipoproteins (BamB-E) of which BamD is essential for growth [31]. In a previous study we identified the small molecule VUF15259 as an inhibitor of BAM-dependent autotransporter secretion [9]. Incubating *E. coli* cells with sub-lethal concentrations of VUF15259 resulted in activation of the σ^E^ stress and Rcs stress responses while the heat-shock response was not induced (Table 1, Figure 3). Consistently, the σ^E^ response is known to be induced by the accumulation of mislocalized OMPs [32] and the novel BAM complex inhibitor darobactin was also shown to provoke both the Rcs and σ^E^ stress responses [33].

To confirm the specificity of the cell envelope stress reporters we analyzed the responses towards known antibiotics with cytosolic targets. As shown in Table 1, antibiotics that target ribosomes (chloramphenicol and tetracyclin), folic acid synthesis (sulphametoxazole), DNA gyrase (levofloxacine) or topoisomerase (nalidixic acid) did neither activate the Rcs nor the σ^E^ or the Cpx responses at the concentrations tested. Levofloxacine and nalidixic acid did induce the heat-shock stress response, consistent with other studies [19,20], likely because of defective chromosomal replication and lethal damage by hydroxyl radicals [34]. Nitrofurantoin did induce the Rcs and heat-shock, but not the σ^E^ or Cpx reporters. It should be noted that the targets of nitrofurantoin are rather undefined [35]. Nitrofurantoin is reduced inside cells to toxic intermediates via a nitroso intermediate. It is believed to cause general oxidative damage thereby affecting cell viability [35]. Possibly, cell envelope components are also sensitive to the toxic reduced intermediate. Finally, exposing *E. coli* cells to A22, an inhibitor of the actin-like protein MreB that plays a role in localizing PG synthesis during elongation, resulted in activation of all stress reporters, except for the Cpx reporter. A22-induced Rcs stress has been reported previously [15,21].

Collectively, using various antibacterial agents we confirmed that the induction/activation of cell envelope stress response systems can conveniently be monitored using our panel of promoter-mNG reporter constructs. Importantly, interference with PG and lipoprotein synthesis, BAM activity and LPS integrity resulted in target-specific reporter profiles, with a predominant activation of the Rcs response system.

### 3.4. Kinetics of Rcs Stress Reporter Induction

Agents that target LPS are known to cause a fast induction of the Rcs response, while stressors with intracellular targets such as PG synthesis or lipoprotein transport show a delayed Rcs response [13,15,21]. To examine whether the P*rprA*-mNG reporter can record such Rcs kinetics we determined the mNG fluorescence and growth of the cells over time in response to the agents described in Table 1. Indeed, reporter fluorescence was already detectable 30 min after addition of the LPS-targeting AMPs (PMB, LL-37, CATH-2 and PMAP-36), peaking after ~2 h (Figure 3A). The transient nature of the induction might indicate that the cells adapt and reduce AMP-mediated damage. Alternatively, the AMPs may have been unstable under the conditions used and lost potency. Interestingly, PMBN showed the same rapid response that, however, did not decline in the time window analyzed. Possibly, the additional effect of PMBN on the IM indirectly affects cell envelope biogenesis. Compound A22, which targets the intracellular protein MreB, also induced a fast albeit moderate Rcs signal that did not decrease in time. This Rcs response towards A22 is consistent with earlier findings [15,21].

As expected, the antibacterial agents with internal targets, ampicillin and mecillinam (PG synthesis [12]), globomycin (lipoprotein transport [16]), VUF15259 (BAM complex activity [9]) and nitrofurantoin (generic oxidative damage [33]), resulted in a much slower generation of the Rcs signal, starting ~90 min after exposure and increasing in the analyzed time frame (Figure 3B).

The observed dichotomy in the fluorescence kinetics suggests that the P*rprA*-mNG construct faithfully reports on Rcs response kinetics.

### 3.5. Differential Kinetics of Rcs Stress Reporter Induction Can Be Exploited in HTS Format

**Figure 4:**
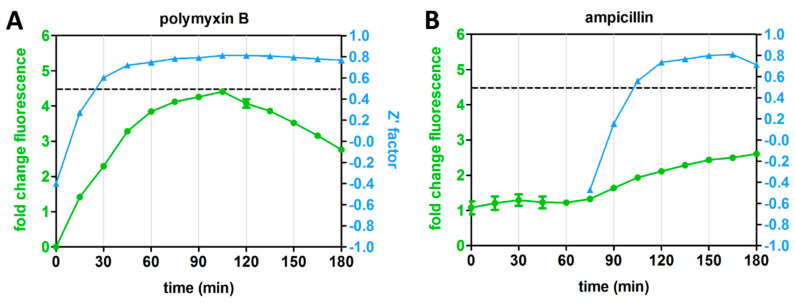

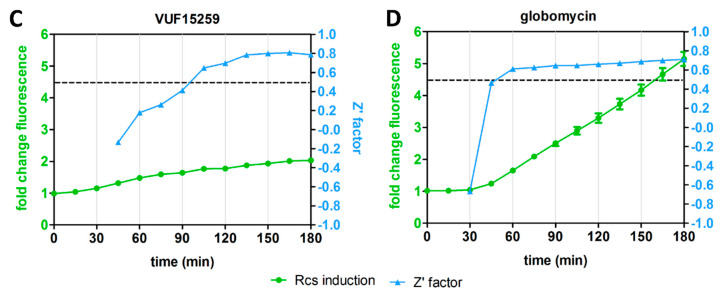
Rcs reporter assay reliability for HTS (high-throughput screening). *E. coli* TOP10F’ cells, harboring the P*rprA*-mNG reporter construct, were grown in a 96-well plate. The cells were either treated with an antibiotic at 0.5× MIC (positive control) or not treated (negative control) in an interleaved-signal format. By measuring the OD_600_-corrected mNG fluorescence plotted as fold-change compared to untreated cells (set to 1), the Z’ factor of the reporter assay was determined at multiple time points for (**A**) polymyxin B (PMB), (**B**) ampicillin (**C**) VUF15259 and (**D**) globomycin. The Z’ factor could not be calculated at early time points for some of the antibiotics (ampicillin, globomycin and VUF15259). The dashed line indicates a Z’ factor of 0.5. Error bars represent the standard deviation of triplicate samples. The figure shows a representative example of three independent experiments.

The distinct kinetics of the Rcs reporter for LPS-binding compounds and inhibitors of cell-envelope biogenesis prompted us to explore whether the assay is robust for target-specific HTS at specific time points. To this end, we determined the Z’ factor for four representative antibiotics with different mechanisms of action and different Rcs stress response kinetics: PMB, ampicillin, VUF15259 and globomycin (Figure 3A,B). The Z’ factor is a tool to evaluate the quality of an assay by informing both on the dynamic range of signal between positive and negative controls and on the data variation [11]. *E. coli* cells containing the P*rprA*-mNG reporter construct were grown in a 96-well plate and exposed to the antibiotics at 0.5× MIC. Fluorescence and growth were followed in time and at each time point the Z’ factor was calculated for cells exposed to each individual agent (Figure 4A–D). A Z’ score >0.5 indicates an outcome that is reliable and discriminative between the positive and negative controls [11]. For PMB, a reliable Z’ factor score of >0.5 (dashed line in Figure 4) was already reached within 30 min of exposure, for globomycin after 45 min, while for ampicillin and VUF15259 at least 105 min of exposure was required. Apparently, measuring the Rcs response at specific timepoints could help in identifying hit compounds for specific targets.

### 3.6. Expression of FhuA ∆C/∆4L Can Support Identification of Large-Scaffold Inhibitors

The OM contains the general porins OmpF and OmpC that facilitate diffusion of hydrophilic or amphiphilic molecules with masses up to approximately 600 Da [1]. Hence, the Rcs assay is limited by this molecular sieve and cannot detect large-scaffold antibiotics (>600 Da) with a target inside the cell envelope. Therefore, vancomycin that acts on PG synthesis and is thus expected to induce Rcs stress cannot be detected because its mass of 1450 Da precludes entry into *E. coli* cells.

**Figure 5:**
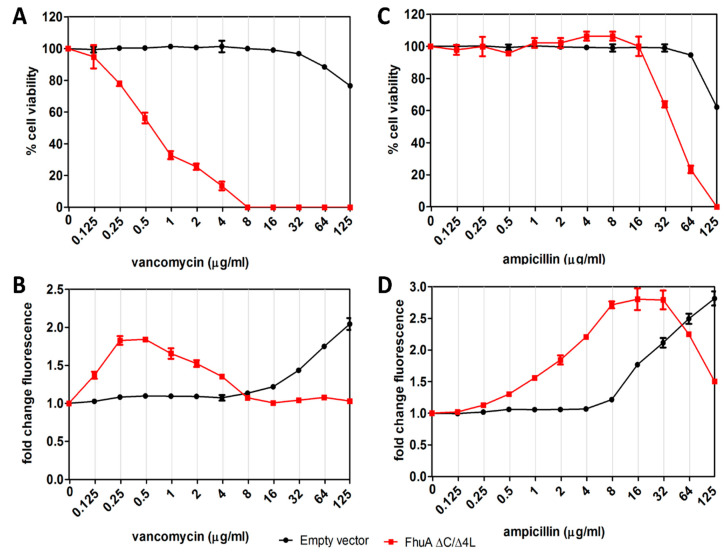
Adapting *E. coli* to detect Rcs stress responses to large-scaffold antibiotics. *E. coli* MC4100 cells, harboring an empty pABCON2 (empty vector) or pABCON2-fhuA ∆C/∆4L plasmid in combination with pUA66-PrprA-mNG were grown in a 96-well plate and exposed to an increasing concentration of (**A**,**B**) vancomycin and (**B**,**D**) ampicillin. Fluorescence was determined 2.5 h after exposure and plotted as OD_600_-corrected fold-change of signal compared to untreated cells (set to 1) as shown in the top panels. The OD_600_-based measurement of cell viability is displayed in the bottom panels, with untreated cells set to 100% cell viability. Error bars represent the standard deviation of duplicate samples. The figure shows a representative example of three independent experiments.

In an attempt to increase the permeability of the *E. coli* OM and thus adapt the assay for larger compounds, we expressed an engineered open channel variant of the OMP FhuA. Native FhuA is a siderophore transporter that forms a 22-stranded β-barrel, which is plugged by the N-terminal domain. Krishnamoorthy and coworkers showed that deletion of the plug domain and four large external loops creates a large pore that is permeable to compounds up to approximately 2000 Da, a cut-off that renders the cells susceptible to killing by vancomycin [36,37]. We then constitutively expressed this engineered FhuA ∆C/∆4L in *E. coli* MC4100 and determined the Rcs stress response using the compatible P*rprA*-mNG reporter construct. As shown in Figure 5A, cells lacking FhuA ∆C/∆4L were affected in viability at concentrations >~61 µg/mL but the range of concentrations and timeframe tested prohibited calculation of the MIC. In contrast, cells expressing FhuA ∆C/∆4L showed a MIC of 7 µg/mL, reflecting the increased permeability of the OM.

As expected for a PG inhibitor, vancomycin also induced Rcs stress (Figure 5B). Concomitant with the decrease in MIC, the concentration of vancomycin inducing the highest level of Rcs stress even decreased ~500-fold upon expression of FhuA ∆C/∆4L. In contrast, the MIC of ampicillin that transfers the OM via porins, is much less affected by expression of FhuA ∆C/∆4L (Figure 5C), although a stronger Rcs stress response is apparent already at lower concentrations (Figure 5D).

In conclusion, the data suggest that cells expressing FhuA ∆C/∆4L are permeable to larger antibiotics, making them a suitable background to identify large-scaffold inhibitors of cell envelope biogenesis.

## 4. Discussion and Conclusions

Previously, we reported on a fluorescence-based phenotypic reporter assay to monitor σ^E^ cell envelope stress and cytosolic heat-shock response in *E. coli* in HTS format for small molecule screening [9]. In the current work, we developed similar assays that monitor the Rcs and Cpx stress response in *E. coli*. By using all four stress reporters we found that interference with specific cellular processes induces a process-specific response profile. Interestingly, compromised cell envelope biogenesis and LPS integrity predominantly activated the Rcs response, which we developed into a robust HTS assay that is suited for phenotypic compound screening.

The Rcs stress reporter was not induced by antibiotics that act in the cytosol and target DNA gyrase (levofloxacin and nalidixic acid), protein synthesis (chloramphenicol and tetracycline) or folic acid synthesis (sulfamethoxazole). In contrast, the PG inhibitors mecillinam and ampicillin, the lipoprotein maturation inhibitor globomycin, the BAM inhibitor VUF15259 and cationic AMPs that target LPS all resulted in strong Rcs induction. This confirms that the Rcs system responds to a wide range of cell envelope stressors, whereas it is rather insensitive to indirect generic stress that may result from antibiotic treatment even at sub-lethal concentrations. Although this specificity was anticipated based on previous studies (see references in Table 1) it has not been shown before in a single comparative analysis.

Similar to Rcs, the Cpx and σ^E^ stress reporters respond to specific cues. Although more antibiotics need to be tested, it appears that specific stress reporters respond to target-specific damage. In contrast, ethanol triggered all responses indicative of generic stress, which is probably related to membrane damage and loss of proton motive force (Table 1 and Figure S1D).

The relative ease of stress-based phenotypic screening argues for the use of stress reporters in HTS assays as a first filter to identify compounds that hit critical targets. The Rcs stress reporter is of particular interest to consider as a primary screen for cell-envelope-specific antibacterials because it responds to various types of PG and OM defects. Importantly, inhibition of underexplored targets such as the BAM complex and lipoprotein trafficking are known [33,38,39] and shown here (Figure 3) to cause Rcs stress.

**Figure 6:**
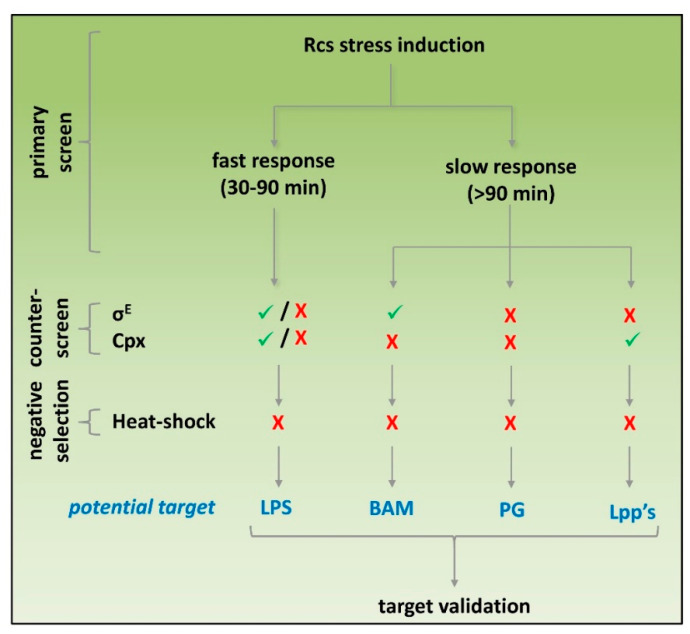
Potential screening strategy to identify cell-envelope-specific antibiotics. Compounds that are tested in a primary screen for Rcs stress activation using P*rprA*-mNG can be categorized into agents that induce a fast Rcs response (detectable within 30–90 min after exposure) or a slow Rcs response (detectable >90 min after exposure). Subsequently, activation of σ^E^, Cpx and heat-shock responses using the P*rpoE*-mNG, P*cpxP*-mNG and P*groES*-mNG reporter constructs, respectively, can be used for counter-screening and subsequent selection of suitable hits. The outcome of the response profiling may give a first indication on the potential targets of hits, which then need to be verified using target-specific approaches.

Real-time monitoring showed that Rcs stress caused by cationic LPS binding peptides could be established with reliable Z’ factors already within 30 min after exposure. In contrast, inhibitors of PG and OM biogenesis show a delayed Rcs response being reliably detected ≥90 min after exposure. The target-specific kinetics of Rcs induction are in line with previous reports (see references Table 1) and would allow us to simultaneously screen libraries for LPS-specific compounds and agents that act within the cell envelope by measuring stress at an early and late time point, respectively. After the primary Rcs stress assay results have been verified, counter-screening for heat-shock stress may exclude compounds that elicit more generic stress. Next, resolving σ^E^ and Cpx stress may help selecting potential targets before engaging in more tedious target-specific molecular and biochemical analysis (Figure 6).

Finally, we present an alternative host strain for the Rcs reporter construct in which the OM is permeabilized by expression of a plug-less FhuA OM pore protein. This allows screening for large-scaffold antibiotics with a molecular mass above the natural sieve size (~600 Da) of the Gram-negative OM [1]. While not useful for Gram-negative bacteria as such, the larger hit compounds identified may affect Gram-positive bacteria if the target is conserved. For example, we show that vancomycin (1450 Da) that kills Gram-positive bacteria by inhibiting PG synthesis can be detected at very low concentrations in the permeable Rcs reporter strain. Furthermore, larger hits may be chemically modified and reduced in size to allow transport across the Gram-negative OM. Alternatively, potentiating compounds (agents that facilitate other compounds to penetrate the cells [40]) can be co-administered or fused to facilitate transfer of the newly identified larger compounds across the OM.

There is increasing interest in the identification and use of these potentiators to broaden the arsenal of antibiotics that can be exploited to combat Gram-negative pathogens. As mentioned, the Rcs reporter in the original host strain responds to LPS-specific potentiators such as the PMB derivative PMBN that, while not being bactericidal by itself, shows OM-permeabilizing activity [28]. Our data suggest that addition of vancomycin to the growth medium at a sub-lethal concentration could make the Rcs HTS also suitable for the selection of potentiators that do not act through interaction with LPS.

## 5. Conclusions

The operationally simple and robust phenotypic Rcs reporter assays described here, combined with counter-screening for σ^E^, Cpx and heat-shock stress, holds promise for HTS analysis of large compound libraries to identify novel classes of cell envelope inhibitors and potentiators.

## Figures and Tables

**Table 1:** Stress response activation by the listed antibacterial agents and their mechanism of action.

				Stress Reporters ^a^			
Antibacterial ^a^	Concentration	Mechanism of Action		Rcs	σ^E^	Cpx	GroES
ampicillin	80 µM	CELL ENVELOPE	inhibit PG synthesis	+ [12]	−	−	−
mecillinam	3 µM			+ [12]	−	−	−
SDS	n.a.		solubilizes lipid bilayer	− [13]	−	−	−
triclosan	n.a.		inhibits fatty acid biogenesis	−	−	−	−
EDTA	40 µM		affects LPS integrity	− [13]	−	+ [14]	−
PMB	0.6 µM		affect LPS integrity/ membrane destabilization	+ [13,15]	−	−	−
PMBN	26 µM			+ [13]	+	+	−
LL-37	10 µM			+ [13]	−	+	−
CATH-2	1.2 µM			+	−	−	−
PMAP-36	1.2 µM			+	−	+	−
VUF15259 [9]	100 µM		inhibits BAM complex	+	+ [9]	−	−
globomycin	38 µM		inhibits lipoprotein maturation	+ [15,16]	−	+ [17]	−
nitrofurantoin	0.6 µM	CYTOSOL	general oxidative damage	+	−	−	+ [18]
levofloxacin	2.7 nM		inhibit DNA synthesis	−	−	−	+ [19]
nalidixic acid	4.3 µM			−	−	−	+ [20]
chloramphenicol	n.a.		inhibit protein synthesis	−	−	−	−
tetracycline	n.a.			−	−	−	−
A22	11 µM		disrupts morphology and chromosome segregation	+ [15,21]	+	−	+
sulfamethoxazole	3.9 µM		inhibits folic acid synthesis	−	−	−	−
ethanol	2.1 nM	GENERAL	generic protein and membrane damage	+	+ [22,23]	+	+ [22,23]

^a^ Plus and minus indicate activation or no activation of the indicated stress responses, respectively. Numbers refer to the reference that confirms activation or absence of a stress response at the indicated concentration (n.a. means no stress was measured). Kinetics of σ^E^, Cpx and heat-shock stress can be found in the Supplementary Materials (Figure S1A–D).

## Supplementary Materials

The following are available online at https://www.mdpi.com/2079-6382/9/11/808/s1, Figure S1: Real-time monitoring of heat-shock, Cpx and σ^E^ stress activation in response to selected antibacterial agents, Table S1: Overview of the strains used in this study, Table S2: Overview of the plasmids used in this study.
