# Supplementary material for: Stress-Based High-Throughput Screening Assays to Identify Inhibitors of Cell Envelope Biogenesis
Source: Antibiotics (Basel). 2020 Nov 13;9(11):808. doi: 10.3390/antibiotics9110808 (PMC7698014; doi:10.3390/antibiotics9110808)
Supplement: Supplementary file 1 [file antibiotics-09-00808-s001.pdf]

Supporting information

Stress-based High-throughput Screening Assays to Identify Inhibitors of Cell Envelope Biogenesis

Maurice Steenhuis[1], Corinne M. ten Hagen-Jongman[1], Peter van Ulsen[1] and Joen Luirink[1,*]

[1]Department of Molecular Microbiology, Amsterdam Institute of Molecular and Life Sciences (AIMMS), Vrije Universiteit, Amsterdam, the Netherlands

*Corresponding author
Joen Luirink, Molecular Microbiology, Vrije Universiteit, De Boelelaan 1085, 1081 HV Amsterdam, the Netherlands. Email: s.luirink@vu.nl. Tel: +31 (0)20 598 7175

Keywords
Escherichia coli, high-throughput screening, antibiotics, potentiators, SigmaE, heat-shock, Cpx, Rcs

Index

Figure S1. Real-time monitoring of heat-shock, Cpx and σ^E stress activation in response to selected antibacterial agents. 3

Table S1. Overview of the strains used in this study. 4

Table S2. Overview of the plasmids used in this study. 4

References 5

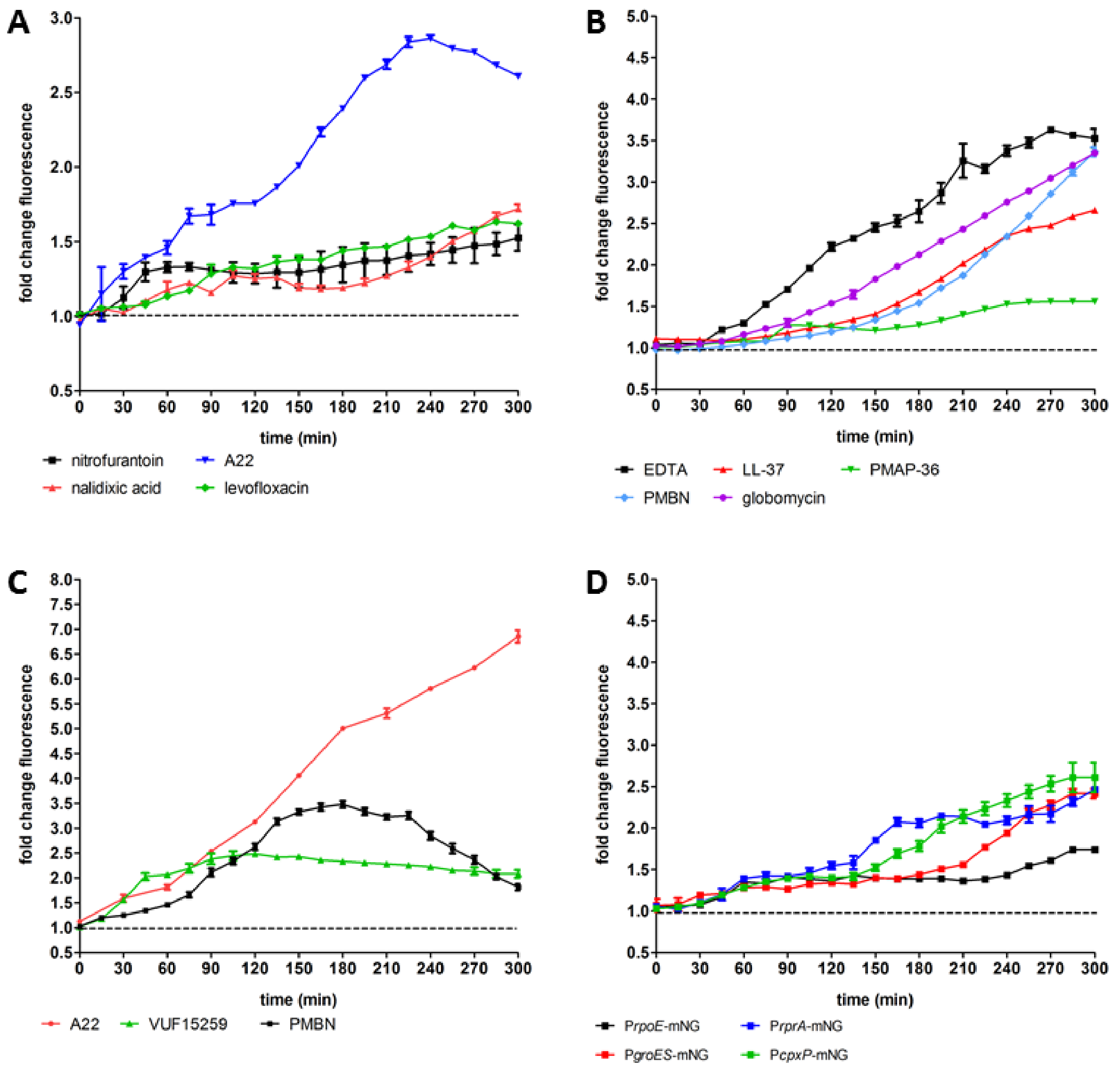

Figure S1. Real-time monitoring of heat-shock, Cpx and σ^E stress activation in response to selected antibacterial agents.

E. coli TOP10F' cells, harboring the P*groES*-mNG, P*cpxP*-mNG or P*rpoE*-mNG reporter constructs to visualize (**A**) heat-shock, (**B**) Cpx and (**C**) σ^E stress respectively, were grown in M9 in a 96-well plate and exposed to 0.5xMIC of the indicated agents. mNG fluorescence was measured in time, corrected for growth (OD_{600}) and plotted as fold-change compared to untreated cells (set to 1, dashed line). (**D**) Cells were grown as described above and σ^E, Rcs, heat-shock and Cpx stress activation was measured in response to 2.5% ethanol using the reporter constructs as indicated, respectively. Error bars represent the standard deviation of duplicate samples. The figure shows a representative example of three independent experiments.

Table S1. Overview of the strains used in this study.

Plasmid	Description	Reference
E. coli TOP10F'	Cloning and expression strain	Thermo Fisher Scientific
E. coli MC4100	Cloning and expression strain	[1]

Table S2. Overview of the plasmids used in this study.

Plasmid	Description	Reference
pUC66-RprA-GFPmut	P_{rprA}-*neongreen*	Matylda Zietek (EMBL)
pBAD22-DjlA	P_{BAD22}-*DjlA*	[3]
pABCON2-fhuA ΔC/Δ4L	P_{OXB11}-*fhuA ΔC/Δ4L*	[4]
pABCON2	Empty vector	[4]
pUA66-RpoE-mNG	P_{rpoE}-*neongreen*	[5]
pUA66-GroES-mNG	P_{groES}-*neongreen*	[5]
pUA66-RprA-mNG	P_{rprA}-*neongreen*	This study
pUA66-CpxP-mNG	P_{cpxP}-*neongreen*	This study
pEH3-Hbp	P_{lac}-hbp	[6]
pEH3-Hbp110C/348C	P_{lac}-hbp110C/348C	[6]

References

(1) Taschner, P. E.; Huls, P. G.; Pas, E.; Woldringh, C. L. Division Behavior and Shape Changes in Isogenic FtsZ, FtsQ, FtsA, PbpB, and FtsE Cell Division Mutants of *Escherichia Coli* during Temperature Shift Experiments. *J. Bacteriol.* **1988**, *170* (4), 1533–1540. https://doi.org/10.1128/jb.170.4.1533-1540.1988.

(2) Baba, T.; Ara, T.; Hasegawa, M.; Takai, Y.; Okumura, Y.; Baba, M.; Datsenko, K. A.; Tomita, M.; Wanner, B. L.; Mori, H. Construction of *Escherichia Coli* K-12 in-Frame, Single-Gene Knockout Mutants: The Keio Collection. *Mol. Syst. Biol.* **2006**, *2*. https://doi.org/10.1038/msb4100050.

(3) Genevaux, P.; Wawrzynow, A.; Zylicz, M.; Georgopoulos, C.; Kelley, W. L. DjlA Is a Third DnaK Co-Chaperone of *Escherichia Coli,* and DjlA- Mediated Induction of Colanic Acid Capsule Requires DjlA-DnaK Interaction. *J. biolo* **2001**, *276* (11), 7906–7912. https://doi.org/10.1074/jbc.M003855200.

(4) Jonkers, T. J. H.; Steenhuis, M.; Schalkwijk, L.; Luirink, J.; Bald, D.; Houtman, C. J.; Kool, J.; Lamoree, M. H.; Hamers, T. Development of a High-Throughput Bioassay for Screening of Antibiotics in Aquatic Environmental Samples. *Sci. Total Environ.* **2020**, *729*, 139028. https://doi.org/10.1016/j.scitotenv.2020.139028.

(5) Steenhuis, M.; Abdallah, A. M.; Munnik, S. M. De; Kuhne, S.; Westerhausen, S.; Wagner, S.; Wel, N. N. Van Der; Wijtmans, M.; *et al.* Inhibition of Autotransporter Biogenesis by Small Molecules. *Mol. Microbiol.* **2019**, *112* (1), 1–18. https://doi.org/10.1111/mmi.14255.

(6) Jong, W. S. P. P.; Ten Hagen-Jongman, C. M.; den Blaauwen, T.; Slotboom, D. J.; Tame, J. R. H. H.; Wickström, D.; de Gier, J.W. W.; Otto, B. R.; Luirink, J.; Jan Slotboom, D.; *et al*. Limited Tolerance towards Folded Elements during Secretion of the Autotransporter Hbp. *Mol. Microbiol.* **2007**, *63* (5), 1524–1536. https://doi.org/10.1111/j.1365-2958.2007.05605.x.
